# Case report: marfan syndrome (MFS) mimicking cutaneous vasculitis

**DOI:** 10.3389/fped.2023.1205255

**Published:** 2023-06-15

**Authors:** Fiona Price-Kuehne, Ebun Omoyinmi, Maha Younes, Matthew Edwards, Despina Eleftheriou, Paul Brogan

**Affiliations:** ^1^ Infection, Immunity and Inflammation Department, University College London Great Ormond Street Institute of Child Health, London, United Kingdom; ^2^ Clinical Genetics and Genomics Laboratory, Royal Brompton Hospital, Guy’s and St. Thomas’ NHS Foundation Trust, London, United Kingdom; ^3^ Rheumatology Department, Great Ormond Street Hospital NHS Foundations Trust, London, United Kingdom

**Keywords:** marfan syndrome, *FBN1* gene, vasculitis, skin rash, aortic dilatation

## Abstract

Marfan syndrome (MFS) is an autosomal dominant connective tissue disorder caused by variants in the extracellular microfibril fibrillin (*FBN1*) gene. Here we report an *FBN1* variant in a child with an unusual skin rash mimicking cutaneous vasculitis, and mild aortic root dilatation. The case was complicated by lack of typical skeletal MFS phenotype; and severe needle phobia preventing any blood testing for workup of suspected vasculitis. Therefore inflammatory markers, autoantibody profile and general hematology/biochemistry results were unknown. Diagnosis of MFS was made via genetic testing of a saliva sample alone using a next-generation sequencing (NGS) targeted gene panel designed to screen for monogenic forms of vasculitis and noninflammatory vasculopathic mimics. This revealed the patient was heterozygous for a pathogenic frameshift variant in *FBN1*; NM_000138, c.1211delC, p.(Pro404Hisfs*44), predicted to cause premature protein truncation leading to loss of function. The variant has not been detected in control populations and has previously been detected in individuals with MFS. This rapid diagnosis significantly impacted the patient management: avoidance of invasive investigations; avoidance of unnecessary immunosuppression; facilitating genetic counselling of the index case and family; and directly informing lifelong monitoring and ongoing treatment for aortic root involvement from MFS. This case further emphasizes the diagnostic utility of NGS early in the diagnostic workup of paediatric patients referred with suspected vasculitis, and we emphasize that MFS can present with cutaneous vasculitic-like features in the absence of the typical Marfanoid skeletal phenotype.

## Introduction

A genetic diagnosis for a child with a rare disease or unusual presentation is often the end point of a “diagnostic odyssey” that has lasted months to years, and involved numerous investigations and invasive procedures. This particularly applies to paediatric patients with suspected systemic vasculitis (1). Such cases are diagnostically challenging since the presenting features are extremely variable, often non-specific, and affect different organ systems resulting in (often fruitless) multispecialty review during the diagnostic journey. Vasculitis of the young is increasingly recognised to be caused by monogenic disorders of the immune system, requiring stratified precision therapies to ensure successful outcomes. Furthermore, it is recognized that non-inflammatory genetic vasculopathies can mimic vasculitis (Table 1), although the treatment for these two groups of pathologies is very different. These points argue strongly for targeted genetic screening using next generation sequencing (NGS) early in the diagnostic workup of children with suspected systemic vasculitis (4, 5).

**Table 1:** Monogenic non-inflammatory vasculopathic mimics of vasculitis of the young.

Diagnosis	Gene/s	Inheritance	Vasculopathic features	Cutaneous features	Other typical features
Marfan syndrome (MFS)	*FBN1*	AD	Aortic root dilatation/dissection Mitral valve prolapse	Striae Atrophic lesions Hyper-elasticity	Pectus excavatum, scoliosis, talipes, hypermobility Spontaneous pneumothoraces Myopia, ectopia lentis
Loeys-Dietz syndrome (LDS)	*TGFBR1* *TGFBR2* *TGFB2* *TGFB3* *SMAD3*	AD	Aortic dissection Cerebral/thoracic/abdominal arterial tortuosity, aneurysm or dissection	Velvety, translucent skin Easy bruising Dystrophic scars Facial milia	Pectus excavatum, talipes Cervical spine instability, craniosynostosis Hypertelorism, strabismus Bifid uvula/cleft palate Intestinal inflammation
Shprintzen-Goldberg syndrome (SGS)	*SKI*	AD	Aortic root dilatation	Minimal subcutaneous fat	Mitral valve prolapse, atrial septal defect Joint hypermobility/contractures, pectus deformity, arachnodactyly Neurodevelopmental delay Craniosynostosis Hirschsprung's disease Facial appearance—hypertelorism, ocular proptosis, downslanting palpebral fissures, micro/retrognathia, tall forehead
Ehlers-Danlos syndrome (EDS)- vascular type	*COL3A1*	AD	Arterial aneurysms or rupture Carotid-cavernous sinus arteriovenous fistula	Thin, translucent skin especially chest/abdomen Acrogeria of extremities Early-onset varicose veins	Talipes, tendon rupture, hypermobility, hip dislocations Intestinal perforation, uterine rupture Facial appearance—thin vermilion, micrognathia, narrow nose, prominent eyes
Familial thoracic aortic aneurysm or dissection (FTAAD) (2)	*ACTA2* *MYH11* *TGFBR2* *MYLK* *PRKG1*	AD	Aortic aneurysm/dissection Ischaemic strokes/cerebral aneurysm Coronary artery disease Abnormal arterial aneurysm	Livedo reticularis May have similar features to LDS/MFS	Iris floculi, patent ductus arteriosus May have similar features to LDS/MFS
Neurofibromatosis Type 1	*NF1*	AD	Renal artery stenosis Aortic dissection	Dermal neurofibromas Café au lait spots Axillary/inguinal freckling	Lisch nodules in the iris Plexiform neurofibromas Schwannomas Optic nerve glioma
Tuberous sclerosis	*TSC1*	AD	Multiple organ system hamartomas Renal angiomyolipomas	Hypomelanotic macules Facial angiofibromas Connective tissue nevi	Epilepsy Learning difficulties
Cerebral autosomal dominant arteriopathy with subcortical infarcts and leukoencephalopathy (CADASIL)	*NOTCH3*	AD	Recurrent strokes Leukoencephalopathy Progressive hypertrophy of vascular smooth muscle in cerebral and extracerebral vessels	N/a	Migraines Cognitive impairment
Cerebral amyloid angiopathy (CAA)	*APP* *CST3* *ITM2B*	AD	Recurrent strokes Progressive amyloid deposition in cerebral blood vessels	N/a	Progressive dementia
Arterial tortuosity syndrome (ATORS)	*SLC2A10*	AR	Widespread tortuosity, elongation, stenosis and aneurysm of the major arteries	Hyperextensible skin	Joint hypermobility/contractures, pectus deformity, arachnodactyly, scoliosis Inguinal and diaphragmatic hernia Facial appearance—micrognathia, elongated face, high palate, beaked nose
Autosomal dominant cutis laxa (ADCL) Autosomal recessive cutis laxa Type 1b (ARCL Type 1b) (3)	*ELN* *FBLN4*	AD AR	Aortic root aneurysm/dissection Arterial tortuosity, elongation and aneurysm and dissection	Wrinkled, inelastic skin Loose skin folds	Emphysematous changes in the lung Facial appearance – long philtrum, large ears, prominent nasolabial folds (less pronounced in ARCL Type 1b) Osteoporosis and easy fracturing

FBN1, fibrillin 1; AD, autosomal dominant; TGFBR1, transforming growth factor beta-receptor 1; TGFBR2, transforming growth factor beta-receptor 2; TGFB2, transforming growth factor beta 2; TGFB3, transforming growth factor beta 3; SMAD3, mothers against decapentaplegic homolog 3; SKI, SK-oncogene; COL3A1, collagen type 3, alpha-1; ACTA2, actin, alpha-2; MYH11, myosin heavy chain 11; MYLK, myosin light chain kinase; PRKG1, protein kinase CGMP-dependent 1; NFI, neurofibromin 1; TSC1, tuberous sclerosis complex subunit 1; NOTCH3, notch receptor 3; APP, amyloid precursor protein; CST3, cystatin 3; ITM2B, integral membrane protein 2B; SLC2A10, solute carrier family 2 member 10; ELN, elastin; FBLN4, fibulin-4.

We present a child with an unusual skin rash and aortic root dilatation referred to our service for workup of suspected large vessel vasculitis. Severe needle phobia had precluded any invasive investigations, including blood tests. In the absence of blood tests, definitive imaging, or biopsies, a genetic diagnosis of Marfan syndrome (MFS) was rapidly obtained using NGS of salivary DNA with a targeted gene panel designed to screen for monogenic forms of vasculitis and non-inflammatory vasculopathic genetic mimics (6).

## Case description

**Figure 1:**
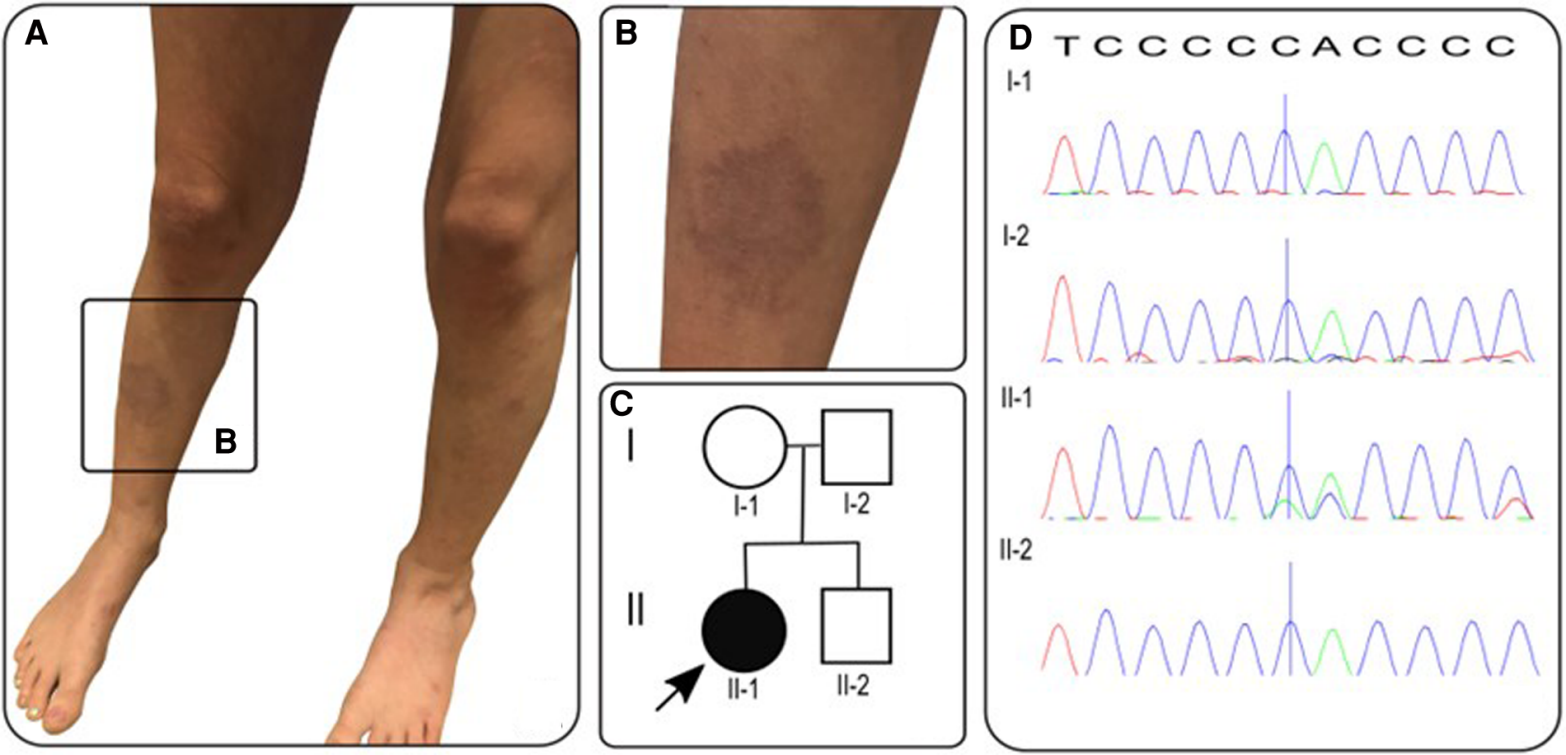
(**A,B**) Photographs of vasculitic-like cutaneous lesions affecting lower legs bilaterally; (**C**) pedigree with proband/affected individual indicated by arrow/black circle; (**D**) sanger electropherograms of *FBN1* gene in family members with blue vertical line indicating deletion of c.1211delC resulting in frameshift mutation in proband (II-1) and reference sequence along top. T, thymine; C, cytosine; A, adenine.

A previously-healthy 10-year-old white-British girl of non-consanguineous parentage was referred to our tertiary pediatric rheumatology service with a 6-month history of worsening skin rash that was suspected to be vasculitic in nature by the local paediatrician. An initial 4 × 2 cm lesion on the right lower leg had gradually increased in size, followed by scattered 1 cm lesions developing across the left leg and buttocks (Figures 1A,B). The macular lesions were asymmetrical with irregular borders and a violaceous color (purpura- like), and there was associated atrophy and subtle telangiectasia with visible vessels. The surrounding skin also had a mottled appearance with mild livedo reticularis-like appearance but was warm to touch with normal capillary refill time. On assessment at our centre, she was systemically well, thriving, with no evidence of systemic vasculitis, specifically: no evidence of granulomatous inflammation in the nasal mucosa; normal blood pressure (93/65) with normal peripheral pulses and no thrills or bruits; normal respiratory and abdominal examination; normal musculoskeletal examination; no evidence of inflammatory eye disease clinically and normal fundi. Dip test of the urine revealed absence of blood or protein.

In the year prior to referral, only limited blood tests were available, revealing no evidence of an inflammatory process with C-reactive protein (CRP) <1 mg/L (reference range 0–20 mg/L) and erythrocyte sedimentation rate (ESR) 2 mm/h (reference range 0–10 mm/h); normal complement C3 and C4 levels; and negative lupus anticoagulant and rheumatoid factor. Her full blood count, renal function, liver function, creatine kinase and lactate dehydrogenase were all within normal limits, as was her urine protein:creatine ratio. There were, however, no recent blood tests available due to severe needle phobia. This significantly affected our ability to assess the patient fully to exclude vasculitis as the cause of the unusual skin rash and for any serological evidence of an ongoing inflammatory or autoimmune process driving vasculitis. However, our clinical assessment was that these findings could also be part of an inherited vasculopathy. Based on that, the pre-test probability of systemic vasculitis was low, and gave us confidence that we had time to work with the child and family to initially avoid invasive investigations in the context of her extreme procedural anxiety.

On further scrutiny of the past medical history, we noted an antenatal history of mitral regurgitation (which had resolved by 30 weeks’ gestation). This had prompted an echocardiogram three months prior to her first clinic appointment, which unexpectedly showed aortic root dilatation, with aortic annulus 18.8 mm (z-score +0.44), sinus of Valsalva 32.2 mm (z-score +3.99), ST junction of 22.9 mm (z-score +2.45) and proximal ascending aorta 23 mm (z-score 2.14). The patient had also previously been investigated for mild hypermobility but had a normal skeletal survey, with no evidence of skeletal dysplasia or other skeletal abnormality, and she had normal arm span/height ratio, with height on the 70th percentile and weight on the 40th percentile.

Specific clinical screening for MFS revealed a systemic score of 0/20, while the median systemic score in patients with MFS is 7/20 (7). However, in recognition of the limitations of the systemic score, especially in childhood, the revised Ghent-2 criteria from 2010 place more emphasis on the cardinal features of ectopia lentis and aortic root dilatation, alongside molecular genetic testing. It is also of note that while skin striae are the only cutaneous manifestation listed in the revised Ghent-2 systemic criteria, other unusual skin rashes like those seen in our case have been reported (8). A skin biopsy or further investigation under general anesthesia was offered but not performed due to the patient's severe procedural anxiety.

With the severe needle-phobia hampering our ability to repeat any blood tests or biopsy the skin lesions, we focused on DNA analysis via a salivary sample using a targeted gene panel of 214 genes, designed to screen for monogenic forms of vasculitis, and genetic vasculopathies that are known to mimic vasculitis of the young, as described previously by our group (6). The full list of genes in this panel are provided as Supplementary Material (Supplementary Table S1). This targeted gene panel revealed a frameshift variant in the fibrillin 1 gene: *FBN1*, NM_000138, c.1211delC, p.P404Hfs*44 het. Sanger sequencing results of parental samples were consistent with the variant having arisen de novo in the proband (Figure 1D). This variant is not listed in the Genome Aggregation Database (gnomAD) (9), there are two entries in ClinVar where submitting laboratories have classified this variant as either pathogenic or likely pathogenic, and the same frameshift caused by a different nucleotide deletion has been previously described in MFS (10). The variant is expected to result in either an abnormal truncated protein product or loss of protein from this allele through nonsense-mediated mRNA decay (11). This allowed us to conclude that this genotype is pathogenic (Class 5) despite lack of experimental functional data directly probing the molecular mechanism of pathogenicity. Loss-of-function variants are a known mechanism in MFS and haploinsufficiency has been associated with a more severe aortic phenotype (12), although both haploinsufficiency and dominant negative effects have been implicated in pathogenesis of MFS (13).

The patient was first seen in our clinic in June 2019 and received the genetic diagnosis in December 2019. During close clinical follow-up until May 2023, no features of systemic vasculitis emerged. In January 2020, due to aortic root dilatation in context of MFS with aortic annulus 19.9 mm (z-score +1.84) and sinus of Valsalva 32.2 mm (z-score +3.75), ST junction 21.83 mm (z-score +1.79) and ascending aorta 22.4 mm (z-score +1.59), she was commenced on treatment with losartan and atenolol at standard doses; the mother reported that this was associated with complete resolution of the cutaneous lesions within three weeks. The patient remained clinically well until February 2022, when she developed recurrent spontaneous pneumothoraces. These were managed with bilateral bullectomy and pleurodesis and to date (May 2023), no other features of MFS have emerged.

## Discussion

MFS is an autosomal dominantly inherited connective tissue disorder caused by mutations in the *FBN1* gene encoding for fibrillin, the major constitutive element of extracellular microfibrils. To date, several thousand variants in *FBN1* have been reported in the literature and the gene is implicated in nine conditions including neonatal MFS and the MASS (mitral, aortic, skin and skeletal) phenotype (14). The MASS phenotype is generally reserved for those cases with progressive mitral valve prolapse but stable aortic dilatation and some consider it the milder end of an MFS spectrum. Diagnosis of MFS in childhood is made even more challenging because clinical manifestations, particularly skeletal and cardiac, are age-dependent and may not yet be present in childhood (15). This challenge is reflected in the revised Ghent-2 criteria that place greater emphasis on molecular genetic results.

Although it is increasingly recognized that vasculitis may be a presenting feature of an ever-expanding list of monogenic autoinflammatory diseases, including deficiency of adenosine deaminase type 2 (DADA2) (16) and STING-associated vasculitis of infancy (SAVI) (17), this case highlights the importance of non-inflammatory vasculopathic mimics of vasculitis of the young, as summarised in Table 1. In our case, in the years since her rapid, and arguably early, molecular diagnosis was obtained, the patient has developed a more typical MFS phenotype, with recurrent pneumothoraces. From a cardiac perspective, she was commenced on atenolol and losartan and has remained asymptomatic with stable aortic dilatation. Ocular examination has also remained normal with no evidence of ectopia lentis.

In summary, we emphasize that cutaneous manifestations of MFS may appear vasculitic in nature and may occur in the absence of typical skeletal features of MFS. Our case also demonstrates that when blood testing is not possible, a molecular diagnosis can be achieved quickly via salivary sample alone. Salivary DNA has proven a useful means of relatively non-invasive genetic testing and can be done remotely, however the main potential downside is the yield of DNA; in one study 45/65 provided adequate DNA concentrations (>50 ng/µl), with only 7 samples providing sub-standard DNA concentration (<25 ng/µl) for diagnostic purposes (18), and this is of particular relevance to paediatric practice (19). An early diagnosis of MFS, which can be difficult to achieve in childhood due to the progressive nature of disease, had significant implications for the patient's subsequent management.

## Data Availability

The original contributions presented in the study are included in the article/**Supplementary Material**, further inquiries can be directed to the corresponding author.

## Supplementary materials

Supplementary Table S1 Full list of genes included in in-house targeted gene panel.
